# Regeneration-associated cell transplantation contributes to tissue recovery in mice with acute ischemic stroke

**DOI:** 10.1371/journal.pone.0210198

**Published:** 2019-01-25

**Authors:** Taira Nakayama, Eiichiro Nagata, Haruchika Masuda, Takayuki Asahara, Shunya Takizawa

**Affiliations:** 1 Department of Neurology, Tokai University School of Medicine, Isehara, Japan; 2 Department of Physiology, Tokai University School of Medicine, Isehara, Japan; 3 Department of Regenerative Medicine Science, Tokai University School of Medicine, Isehara, Japan; University of South Florida, UNITED STATES

## Abstract

Various cell-based therapeutic strategies have been investigated for vascular and tissue regeneration after ischemic stroke. We have developed a novel cell population, called regeneration-associated cells (RACs), by quality- and quantity-controlled culture of unfractionated mononuclear cells. RACs were trans-arterially injected into 10-week-old syngeneic male mice at 1, 3, 5 or 7 days after permanent middle cerebral artery occlusion (MCAO) to determine the optimal timing for administration in terms of outcome at day 21. Next, we examined the effects of RACs injection at day 1 after MCAO on neurological deficits, infarct volume, and mediators of vascular regeneration and anti-inflammation at days 7 and 21. Infarct volume at day 21 was significantly reduced by transplantation of RACs at day 1 or 3. RACs injected at day 1 reduced the infarct volume at day 7 and 21. Angiogenesis and anti-inflammatory mediators, VEGF and IL-10, were increased at day 7, and VEGF was still upregulated at day 21. We also observed significantly enhanced ink perfusion *in vivo*, tube formation *in vitro*, and definitive endothelial progenitor cell colonies in colony assay. These results suggest that RAC transplantation in MCAO models promoted significant recovery of neural tissues through intensified anti-inflammatory and angiogenic effects.

## Introduction

Recent treatments for acute ischemic stroke are mostly focused on vascular recanalization, including interventional treatments and intravenous thrombolysis, which have a narrow therapeutic time windows after onset. Therefore, these treatments have only benefited relatively small numbers of stroke patients. Edaravone is also available as a brain-protective therapy in the acute phase of ischemic stroke, but has so far been approved only in Japan. [1] On the other hand, pioneering approaches using embryonic stem cells [2] and induced pluripotent stem cells [3] have been devised, and there is increasing preclinical and clinical evidence that transplantation of specific somatic stem cells or progenitor cells, such as endothelial progenitor cells (EPCs), can promote recovery from ischemic cerebral injury. [4–10]

EPCs were first isolated from peripheral blood (PB) of adults by Asahara and colleagues in 1997 [11]. Circulating EPCs derived from bone marrow were shown to contribute to postnatal physiological and pathological neovascularization [12, 13], which is consistent with a role in vasculogenesis. With the aim of obtaining EPCs-enriched cell populations for clinical application with simple and economical methodology, we have recently developed a novel cell population, named regeneration-associated cells (RACs), by means of quality- and quantity-controlled culture of unfractionated mononuclear cells (MNCs) [14, 15] in the presence of human recombinant stem cell factor (SCF), thrombopoietin, Flt-3 ligand, vascular endothelial growth factor (VEGF) and interleukin-6 (IL-6).

The methodology of this vasculogenic conditioning control of MNCs is both simple and safe, and can be used not only to enhance EPC expansion, but also to activate anti-inflammatory and angiogenic monocytes/macrophages and helper T lymphocytes, resulting in the delivery of various protective and proangiogenic cytokines and growth factors.[14, 15] Thus, increased EPCs and anti-inflammatory monocyte/macrophages in RACs are expected to contribute to the regenerative process through anti-inflammatory and angiogenic signals in ischemic stroke.

Based on the above findings, we speculated that the delivery of RACs during the acute phase of cerebral ischemia might ameliorate the impact of severe ischemia and inflammation, and promote recovery of cerebral infarction patients. To test this idea, we have conducted a preclinical study of RACs transplantation in murine permanent middle cerebral artery occlusion (MCAO) models.

## Materials and methods

### Animals

**Fig 1:**
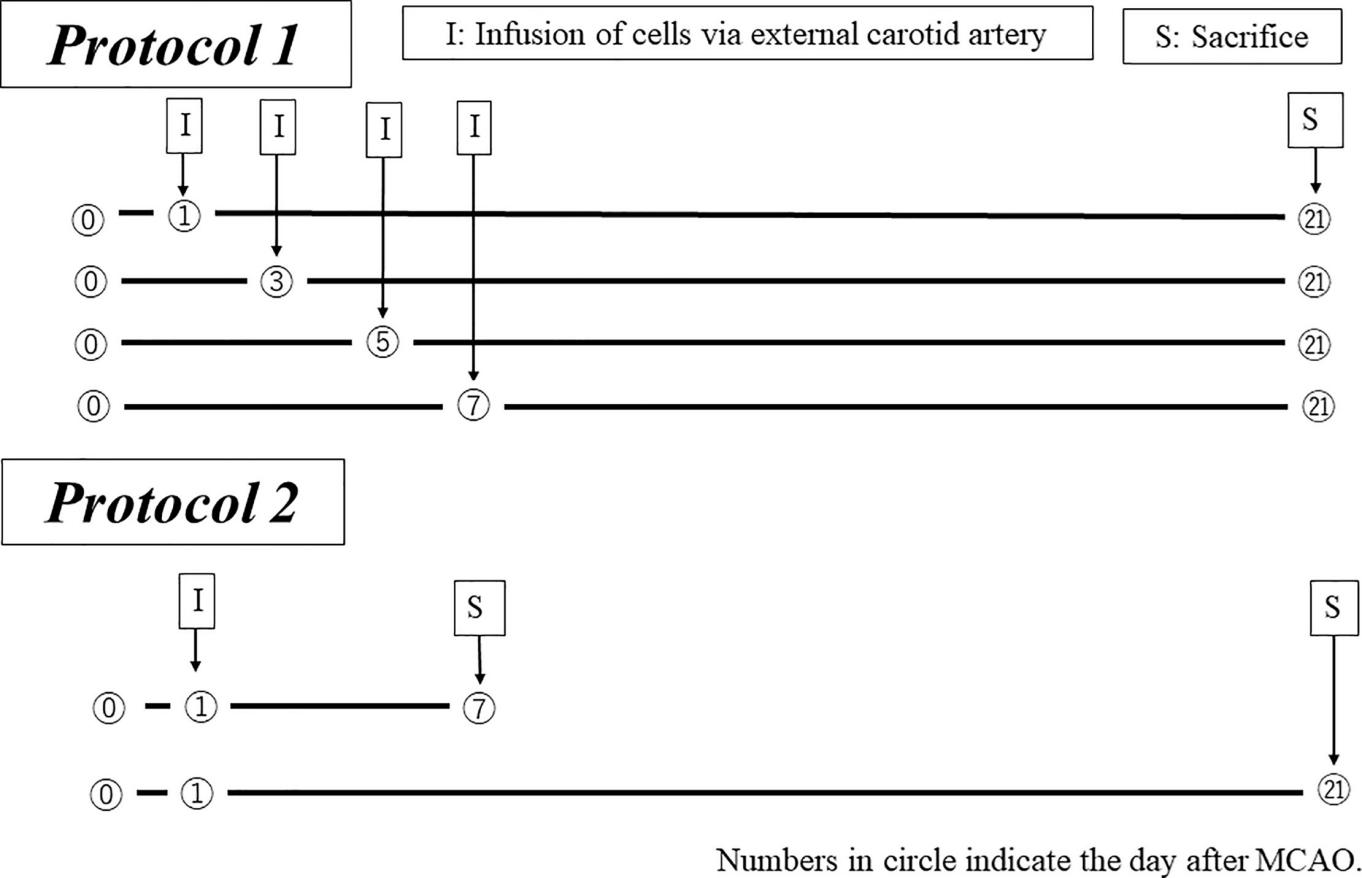
Experimental protocols (transplantation of MNCs and RACs). Protocol 1: Transplantation of MNCs and RACs. Cells were injected at various timings (day 1 after middle cerebral artery occlusion (MCAO) (PBS: n = 13, MNCs: n = 12, RACs: n = 13); day 3 (PBS: n = 8, MNCs: n = 8, RACs: n = 7) after MCAO; day 5 (each group: n = 5) after MCAO; day 7 (each group: n = 5) after MCAO). At day 21 after MCAO, brains were excised and infarct volumes were determined. Protocol 2: Based on the results of Protocol 1, day 1 after MCAO was selected as the best timing for transplantation of MNCs and RACs. Brains were excised and evaluated at day 7 (each group: n = 9) and day 21 (PBS: n = 8, MNCs: n = 7, RACs: n = 9) after MCAO.

C57BL/6J male mice (10 weeks old, weighing 23–25 grams) were purchased from Japan CLEA (Tokyo, Japan). All experimental procedures and protocols were approved by the Animal Care and Use Committee of Tokai University School of Medicine (approval #12R-068). All mice were checked every day. We provided ad libitum access to food and water under controlled lighting in ventilated cages with soft wood chip bedding, with each cage containing five to seven mice. The mice were anesthetized with 4% isoflurane /66% N_2_O /30% O_2_ and maintained with 1.5% isoflurane in all experiments. A total of 234 mice were used in this study, of which four died during or after surgery. Cervical dislocation was performed with anesthetized mice by experienced technician at the endpoint in protocol 1 and 2 (Fig 1).

### Cell culture system

PB (800 μL per mouse) was drawn from the left ventricle of the heart. PB-MNCs were isolated by density gradient centrifugation using Histopaque-1083 (d = 1.083; Sigma, St Louis, MO, USA), and RACs were obtained by culture for 5 days at the cell density of 5 x 10^5^ cells/500 μL in a defined serum-free medium (S0192-500ML StemLineII; Sigma), which contained 5 mouse recombinant proteins (SCF #300–07, TPO #300–18, Flt-3 ligand #300–19, VEGF #100–20 and IL-6 #200–06, PeproTec), on a 24-well Primaria tissue culture plate (5 x 10^5^ cells/500 μL per well (BD Falcon; BD Biosciences, USA)), as previously reported. [14, 15] After 5 days of culture, non-attaching cells were removed by washing with PBS, and adherent cells were harvested with 2 mmol/L of EDTA/PBS. Harvested cells were suspended in Iscove’s modified Dulbecco’s medium (IMDM) (Sigma Aldrich), at a density of 1 x 10^4^ cells/50 μL.

### EPC-colony forming assay (EPC-CFA)

To investigate vasculogenic potential, cells were cultured in semisolid medium (M3236 MethoCult SFBIT; Stemcell Technologies Inc., Vancouver, BC, Canada) with proangiogenic growth factors/cytokines, as previously reported, on 35-mm Primaria dishes (BD Falcon; BD Biosciences, USA) and then adhesive EPC colonies were counted. [13] Aliquots of the cells were seeded at 2 x 10^5^ cells/dish (3 dishes per PB-MNC/ RACs) for EPC-CFA. Ten to 12 days after initiation of culture, the number of adherent colonies per dish was measured using a gridded scoring dish (Stemcell Technologies, Vancouver, Canada) under a phase-contrast light microscope (Eclipse TE300; Nikon, Tokyo, Japan).

### Induction of focal cerebral ischemia

Anesthesia was induced in mice with 4% isoflurane /66% N_2_O /30% O_2_ and maintained with 1.5% isoflurane. Permanent focal ischemia was achieved as follows: a 2-mm hole was drilled at a site superior and lateral to the left foramen ovale to expose the left middle cerebral artery (MCA). The proximal portion of the left MCA was permanently occluded over a 1 mm segment distal to the origin of the lenticulostriate branches using a bipolar coagulator. [16] During occlusion, mice were kept in a humidity-controlled, 32°C chamber to help maintain a body core temperature of 37°C. The incision was sutured, and the mice were again placed in the humidity- and temperature-controlled chamber for another 2 hours and finally returned to their cages for experiments.　

After MCAO, the cells were delivered from the ipsilateral external carotid artery to the internal carotid artery in a retrograde manner. As soon as the cells were injected, the external carotid artery was ligated to avoid leakage of cells. Two experimental protocols were employed, as shown in Fig 1. Protocol 1 was designed to determine the appropriate timing for RACs administration in terms of the outcome at day 21 after occlusion. At days 1, 3, 5, and 7 after occlusion of MCA, we injected PBS (control), MNCs, or RACs into the ipsilateral external carotid artery, and measured the infarct volume at day 21. The dose of RACs for injection was adjusted to 1.0 x 10^4^ /mouse; this was the same as in the previous hindlimb ischemia studies.[13, 15] Previous EPCs transplantation studies for ischemic stroke had used 0.5–1.0 x 10^6^/mouse, [17, 18] since that was the same as the dose used for ischemic hindlimb studies with EPCs. [19, 20] In Protocol 2, RAC transplantation was done at day 1, based on the results obtained with Protocol 1. Protocol 2 was designed to evaluate neurological deficits and infarct volume, as well as to examine changes of mediators of vascular regeneration and anti-inflammation, at days 7 and 21 after MCAO. Animals were randomly divided into each experimental group in protocol 1 and 2, in a blinded manner.

### Evaluations of neurological deficits, relative cerebral perfusion and infarct volume

Neurological deficits were evaluated in terms of neurological grading scores from 0 to 3, depending upon severity [21], including forelimb flexion, resistance to lateral push and circling behavior, at 1 hour and at days 7 and 21 after MCAO. Relative cerebral perfusion was measured by laser-Doppler flowmetry (moorFLPI, Moor Instruments Ltd., UK) at 5 min before and after occlusion, and at day 7 or 21 after occlusion.

For the estimation of infarct volume, the brain was removed and 1-mm coronal sections were cut, stored in 4% paraformaldehyde, stained with hematoxylin and eosin, and photographed. Infarct areas in the cerebral cortex and striatum were measured using NIH Image, and infarct volume (mm^3^) was calculated by multiplying each area by the distance between sections, by an examiner (E. N.) who was blinded as to the animal’s experimental status.

### Immunohistochemistry of factors relating to angiogenesis and anti-inflammation in tissues

At day 7 or 21 after MCA occlusion, mice were briefly re-anesthetized and sacrificed. The brains were removed and cut into 1-mm-thick coronal sections. Coronal brain sections fixed with 4% paraformaldehyde were incubated in 5 mM hydrogen peroxide for 10 min and then exposed to 5% normal goat serum for 10 min. For immunostaining of eNOS and iNOS, the sections were incubated overnight with rabbit anti-rat eNOS antibody (Enzo Life Science, New York, USA) and anti-iNOS antibody (Cell Signaling, MA, USA) at 100-fold dilution, respectively. The sections were then incubated at room temperature for 1 hour with biotinylated anti-mouse IgG (Vectastain Elite ABC peroxidase kit), followed by ABC reagent for 30 min. Bound antibody was visualized with 3,3’-diaminobenzidine and hydrogen peroxide. For immunostaining for VEGF, IL-10 and Iba-1, the sections were first incubated overnight with polyclonal anti-goat VEGF (AF493NA; R&D Systems, MN, USA) at 50-fold dilution, monoclonal anti-rat IL-10 (abcam#ab33471, Cambridge, UK) at 200-fold dilution, and polyclonal anti Iba-1(WAKO, Osaka, Japan) at 1000-fold dilution, respectively, in a humidified chamber at 4°C. The sections were washed thoroughly with 0.01 M sodium phosphate-buffered saline (pH 7.2), then incubated with Histofine Simplestain Mouse Max PO (Nichirei, Tokyo, Japan) at 100-fold dilution for 1 hour. The bound antibody was visualized with 3,3’-diaminobenzidine and hydrogen peroxide. Cells positive for iNOS, eNOS, VEGF, IL-10, and Iba-1 in peri-infarct regions of the frontal cortex and striatum, which were defined as regions within 0.6 mm from the edge of the ischemic region based on hematoxylin and eosin staining, were counted by one examiner (E.N.) blinded as to the experimental protocol, in each of 3 predetermined areas (0.62 mm^2^) per high-power field (x 400).

### Expression profiles of factors relating to angiogenesis and anti-inflammation in tissues

Mouse brain hemisphere was sonicated in cell lysis buffer (50 mM Tris–HCl [pH 7.4], 1% Triton X-100, 0.5 mM PMSF, 2 mM CaCl_2_, proteinase cocktail), and protein concentration was determined using a Protein Assay Kit (Bio-Rad Laboratories, CA, USA) with bovine serum albumin as a standard. The samples were then separated by gel electrophoresis on a 4–12% gradient. After electrophoretic transfer to a polyvinylidene fluoride (PVDF) membrane (Immobilon-P; Millipore, MA, USA), the membrane was blocked with 4% bovine serum albumin in PBS, washed, and incubated overnight with the primary antibodies at 4˚C. Then, the membranes were washed with PBS-T (0.1% Tween20) and incubated with appropriate horseradish peroxidase-conjugated secondary antibodies (Vector Laboratories, CA, USA) for 2 hours at room temperature. The membranes were examined using an enhanced chemiluminescence western blotting system (Amersham-Pharmacia, NJ, USA). In the present study, we used primary antibodies against eNOS (Enzo Life Sciences Inc. NY, USA), iNOS (Cell Signaling Technology Inc., MA, USA), IL-10 (Abcam plc, Cambridge, UK), and VEGF (R&D Systems Inc., MN, USA). Equal protein loading was confirmed using β-actin antibody (Sigma-Aldrich, MO, USA).

### Matrigel tube formation assay

The standard Matrigel method was used as a surrogate in vitro angiogenesis assay to assess the spontaneous formation of capillary-like structures of seeded vascular cells. A standard 96-well plate was coated with 50 μL of Matrigel per well and incubated at 37°C for 15 min. Then, 3 x 10^3^ cells/50 μL PB-MNC and RACs mixed with HUVEC (1.5 x 10^4^ cells/each well) were seeded into each well in the plate, and 100 μL/well Matrigel was added. The plate was incubated at 37°C for 4 hours. The degree of tube formation was determined by counting the number of vascular circles in each well under a 40 x objective. The experiments were performed in triplicate.

### Visualization of arteries with urethane resin

In a separate set of animals (n = 7 in each group), arteries at the brain surface were visualized at day 7 after occlusion, according to Yukami et al. [22] with some modifications. Under deep pentobarbital anesthesia, the right atrium of the heart was incised to allow for venous outflow, the left ventricle of the heart was cannulated, and 2 mL saline was injected. Immediately after the saline injection, 0.5 mL white urethane resin mixed with 50 μL/mL black pigmented ink (Hitohada gel, Exseal Co., Ltd. Japan) was injected. The brain was carefully removed and the top of the brain was photographed at x 50 magnification. The number of arteries was counted in a pre-determined belt area located at 0.6 mm width from the edge of the terminal portion of MCA territory in the border zone with the anterior cerebral artery or posterior cerebral artery. The measurements were made by one individual (E. N.), who was blinded as to the animal treatment group.

### Statistical analysis

Statistical comparisons were made using GraphPad Prism6 (GraphPad Software Inc., San Diego, CA, USA). The Kruskal-Wallis test was used for more than three groups and the Mann Whitney test for two groups. The criterion of significance was a p value of 0.05 or less. Data are presented as mean ± SE.

## Results

### Determination of transplant timing of RACs into MCAO mice

**Fig 2:**
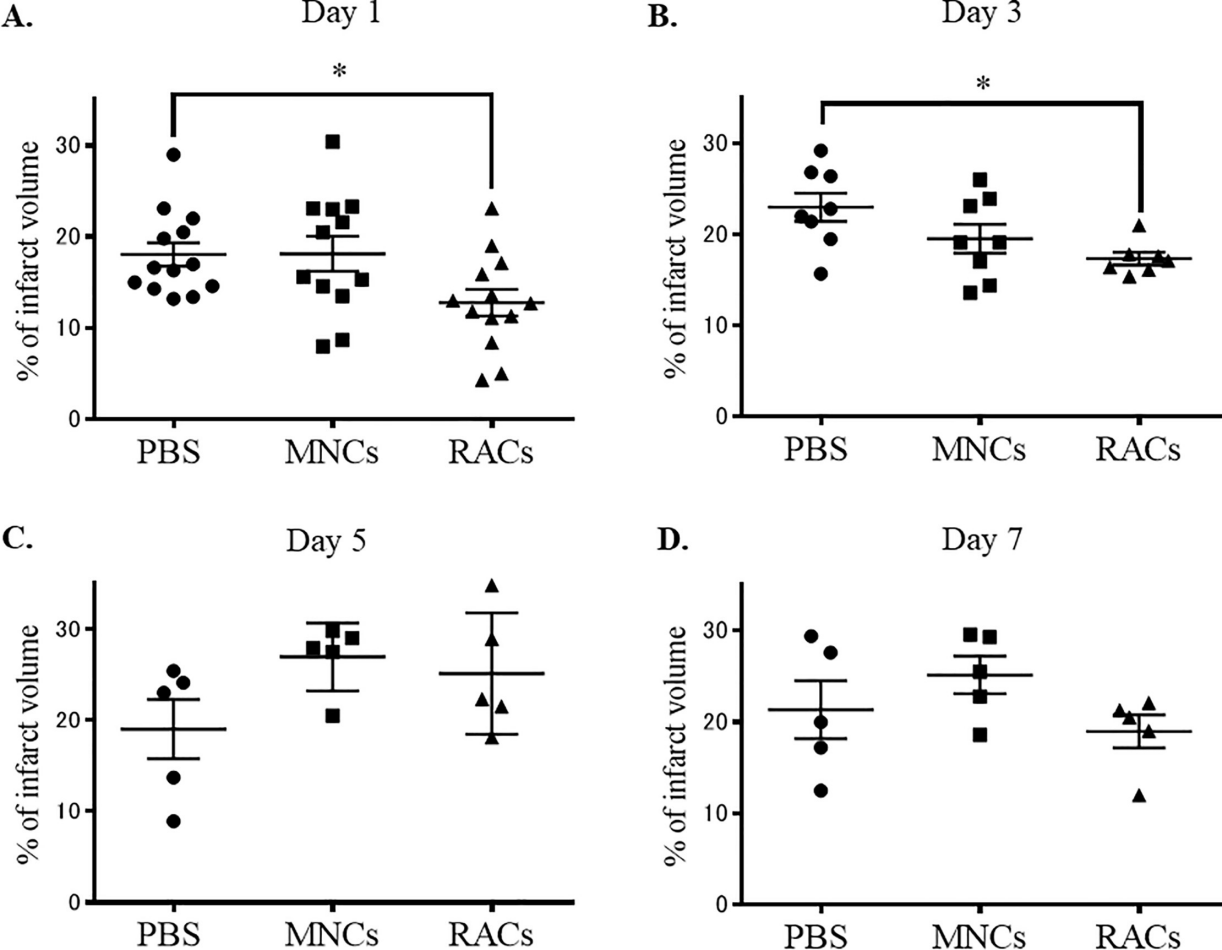
Infarct volumes at day 21 following various cell transplantation timings (Protocol 1). There was a significant difference (*P<0.05, Kruskal-Wallis test) in infarct volume between the PBS injection and RAC injection groups at day 1, whereas PB-MNC had no effect (A) (PBS: n = 13, MNCs: n = 12, RACs: n = 13). Similar findings were observed in animals transplanted at day 3, showing a significant effect of RAC injection but not of MNC injection, compared to the PBS control group (B) (PBS: n = 8, MNCs: n = 8, RACs: n = 7). There was no difference in infarct volume between PBS and RACs (1 x 10^4^ /50 μL) in the case of cell transplantation at day 5 (C) (each group: n = 5) or at day 7 (D) (each group: n = 5) after MCAO.

**Fig 3:**
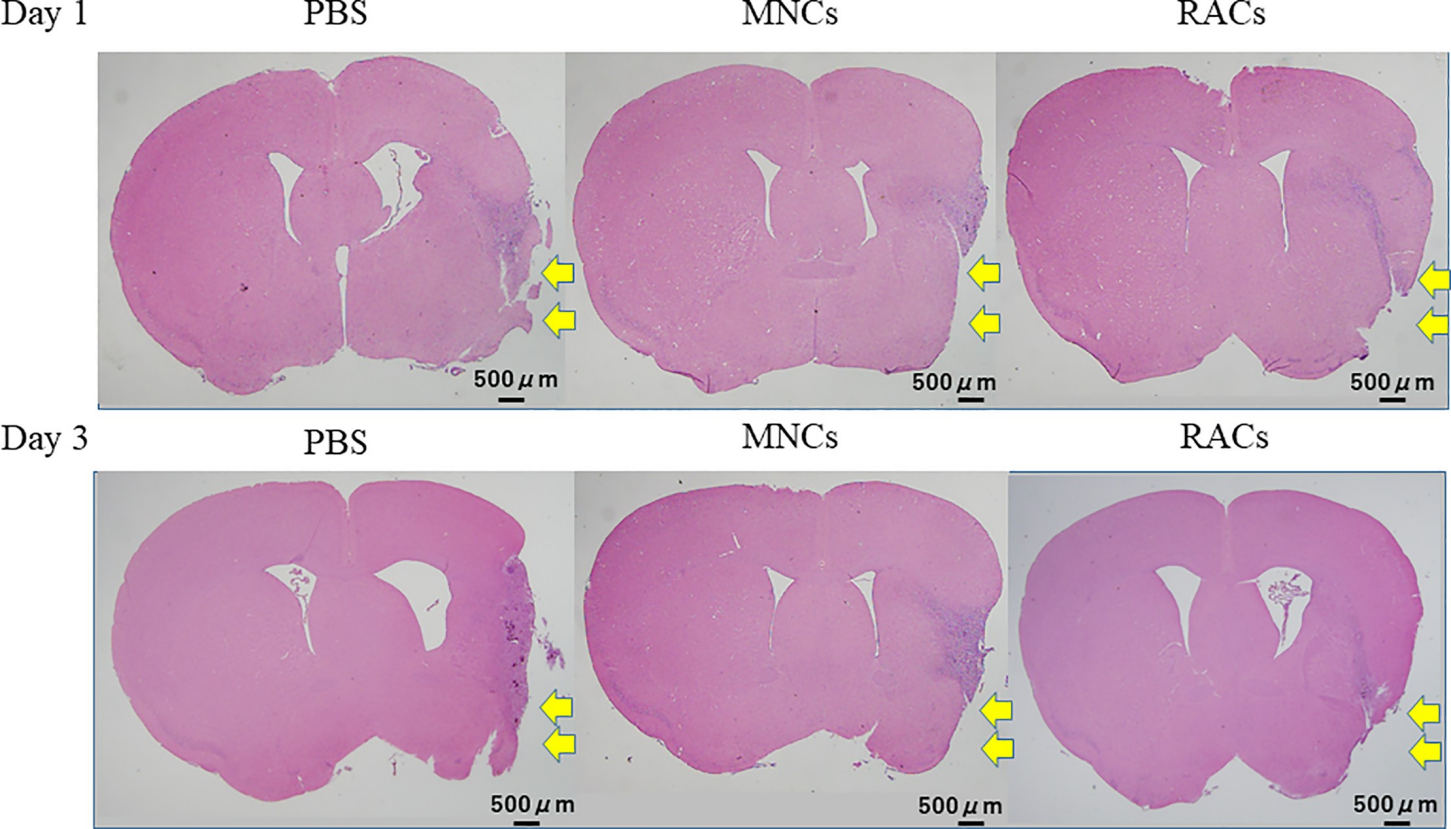
Representative pictures of hematoxylin and eosin staining in mice transplanted at day 1 and 3, and sacrificed at day 21. Yellow arrows showed infarct regions that disintegrated or shrank due to cell clearance, including necrosis, phagocytosis, and gliosis. RACs transplantation at day 1 and 3 reduced the infarct volume, determined by integrating the H&E-stained areas, comparing to PBS injection. Scale bar: 500 μm.

Protocol 1 was designed to determine the appropriate timing for administration of RACs, based on the outcome at day 21 after occlusion. Either decreasing the infarct volume or improving the neurological score was the end point for this protocol. In Protocol 1, RACs injected into mice at day 1 (PBS: n = 13, MNCs: n = 12, RACs: n = 13) and day 3 (PBS: n = 8, MNCs: n = 8, RACs: n = 7) significantly reduced the infarct volume (P<0.05, Kruskal-Wallis test) compared with the PBS group, while PB-MNC showed no effect (Fig 2A and 2B; the results of hematoxylin and eosin staining are shown in Fig 3). However, transplantation of RACs at day 5 (each group: n = 5) and day 7 (each group: n = 5) had no significant effect on infarct volume (Fig 2C and 2D). (Protocol 1). The Bederson scores, representing neurological deficits, and physical parameters are summarized in Table 1. There was no significant difference in Bederson score, body weight, body temperature or CBF among the PBS-, MNCs-, and RACs-treated groups at any cell administration timing after MCAO. Based on these results, we identified day 1 and day 3 as suitable timings for RACs inoculation in subsequent experiments. For experimental convenience, day 1 transplantation was selected as the standard timing to investigate the effects of RACs on MCAO models.

**Table 1:** Physiological parameters (protocol 1).

		Bederson score			Weight(gram)			Temperature(°C)		
Day of injection		PBS	MNCs	RACs	PBS	MNCs	RACs	PBS	MNCs	RACs
Day 1	Pre-operation	15±0	15±0	15±0	23.95±1.60	23.53±1.58	24.45±1.52	31.52±8.46	30.27±9.07	32.70±7.45
	Injection	11.9 ±2.0	11.5 ±2.5	12.8 ±1.8	27.82±8.29	28.15±8.37	26.68±7.14	31.75±7.27	31.24±7.79	33.77±6.17
	Sacrificed	14.3±1.1	14.3±0.9	14.8±0.4	28.20±7.48	29.62±6.82	28.28±6.08			
Day 3	Pre-operation	15±0	15±0	15±0	24.54±1.72	24.84±1.12	24.63±0.99	23.38±1.74	23.50±1.39	23.03±1.16
	Injection	13 ±1.2	12.3 ±1.9	13.4 ±0.8	37.83±0.69	37.76±0.40	37.80±0.62	24.74±1.56	25.43±0.96	23.46±2.84
	Sacrificed	14.0 ±1.4	14.1 ±1.1	13.4 ±1.1	37.21±0.51	37.66±0.42	37.60±0.36			
Day 5	Pre-operation	15±0	15±0	15±0	24.32±1.54	25.28±1.56	22.82±0.84	24.76±1.20	25.32±1.75	23.42±0.76
	Injection	14±0	13.6 ±0.5	13.8 ±0.4	37.74±0.18	37.48±0.59	37.32±0.78	26.30±1.72	25.82±2.04	25.14±1.69
	Sacrificed	14.4 ±0.9	15±0	14.6 ±0.5	37.42±0.55	37.72±0.36	37.60±0.37			
Day 7	Pre-operation	15±0	15±0	15±0	25.06±1.61	25.22±1.37	22.82±0.80	23.74±1.74	24.00±1.42	22.78±0.67
	Injection	11.0 ±2.4	11.4 ±2.1	12.0 ±0.1	37.48±0.68	38.42±0.54	38.08±0.41	24.26±2.09	24.26±1.89	23.78±1.05
	Sacrificed	14.8 ±0.4	14.2 ±0.4	14.6 ±0.6	36.78±0.69	38.00±0.37	37.16±0.54			

There was no significant difference between animals injected with PBS and MNCs or RACs, injected on either day 1 (PBS: n = 13, MNCs: n = 12, RACs: n = 13), day 3 (PBS: n = 8, MNCs: n = 8, RACs: n = 7), day 5 (each group: n = 5), or day 7 (each group: n = 5) in terms of Bederson score at the late phase, weight at any time, and temperature at any time (pre-operation, injection, and scarification).

### Effect of RAC transplantation on cerebral infarction after MCAO

**Fig 4:**
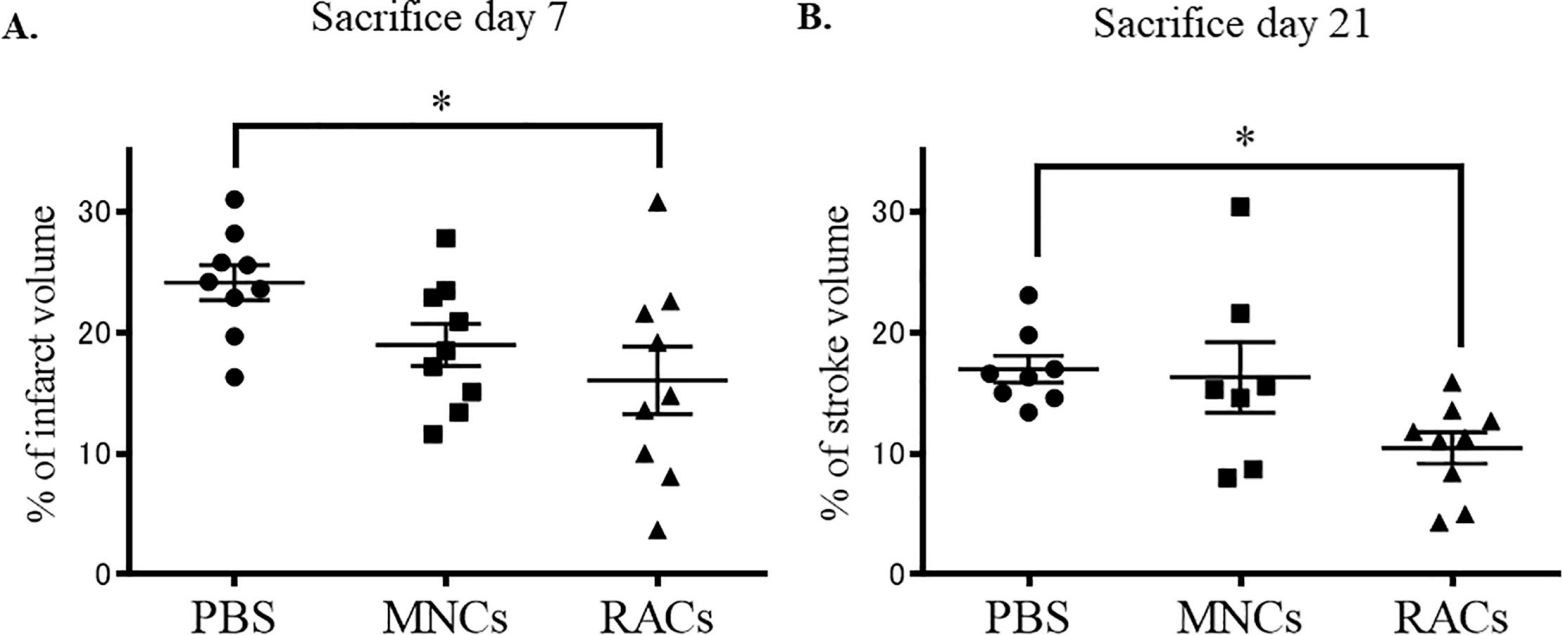
Infarct volume at day 7 (early phase) and day 21 (late phase) after cell transplantation. Injection of RACs (1 x 10^4^ /50 μL) at day 1 significantly (*P<0.05, Kruskal-Wallis test) reduced the infarct volume compared with the PBS group, at both day 7 (A) (each group: n = 9) and day 21 (B) (PBS: n = 8, MNCs: n = 7, RACs: n = 9) after MCAO, while PB-MNCs had no effect.

Protocol 2 was designed to examine the effects of day 1 transplantation on tissue biology in cerebral infarction at day 7 and day 21 (early and late phase, respectively) after MCAO (Fig 1). We evaluated the physical findings of the neurological deficits and the infarct volume, as well as the tissue expression profiles of angiogenesis and anti-inflammatory mediators using immunohistochemistry and western blotting. As shown in Table 2, there was no significant difference at either early or late phase follow-up between animals given PBS, MNCs and RACs in terms of Bederson score, weight, or temperature at pre-operation, injection, or scarification. However, RACs injected at day 1 into mice significantly reduced the infarct volume (P<0.05, Kruskal-Wallis test) compared with PBS when tissue deficits were evaluated at both day 7 and day 21, though PB-MNCs had no effect (Fig 4).

**Table 2:** Physiological parameters (protocol 2).

		Bederson score			Weight(gram)			Temperature(°C)		
		PBS	MNCs	RACs	PBS	MNCs	RACs	PBS	MNCs	RACs
Early phase	Pre-operation	15±0	15±0	15±0	24.42 ±1.02	24.01 ±0.80	23.47 ±0.80	37.76 ±0.31	37.40±0.32	37.81±0.70
	Injection	10.6 ±2.7	11.7 ±1.9	11.3 ±2.1	21.63 ±1.04	21.0 ±1.30	21.16 ±0.82	37.3 ±1.51	36.33±0.71	36.80±0.34
	Sacrificed	13±0.9	12.8 ±2.0	12.1 ±1.8	22.27 ±1.35	21.61 ±1.90	21.07 ±2.86			
Late phase	Pre-operation	15±0	15±0	15±0	24.53 ±1.39	24.34 ±1.09	24.5 8±1.79	37.89 ±0.67	37.53 ±0.69	37.44 ±0.81
	Injection	11±2.0	10.2 ±2.2	12.1 ±1.8	21.55 ±0.92	21.39 ±0.55	22.14 ±1.12	37.16 ±0.57	37.41 ±0.49	37.69 ±0.79
	Sacrificed	14.3 ±1.4	14.1 ±0.9	14.9 ±0.3	22.81 ±3.08	24.14 ±1.12	24.48 ±1.54			

There was no significant difference between PBS and MNCs or RACs at either the early (each group: n = 9) or the late phase (PBS: n = 8, MNCs: n = 7, RACs: n = 9) in terms of Bederson score, weight, or temperature at pre-operation, injection, or scarification.

**Fig 5:**
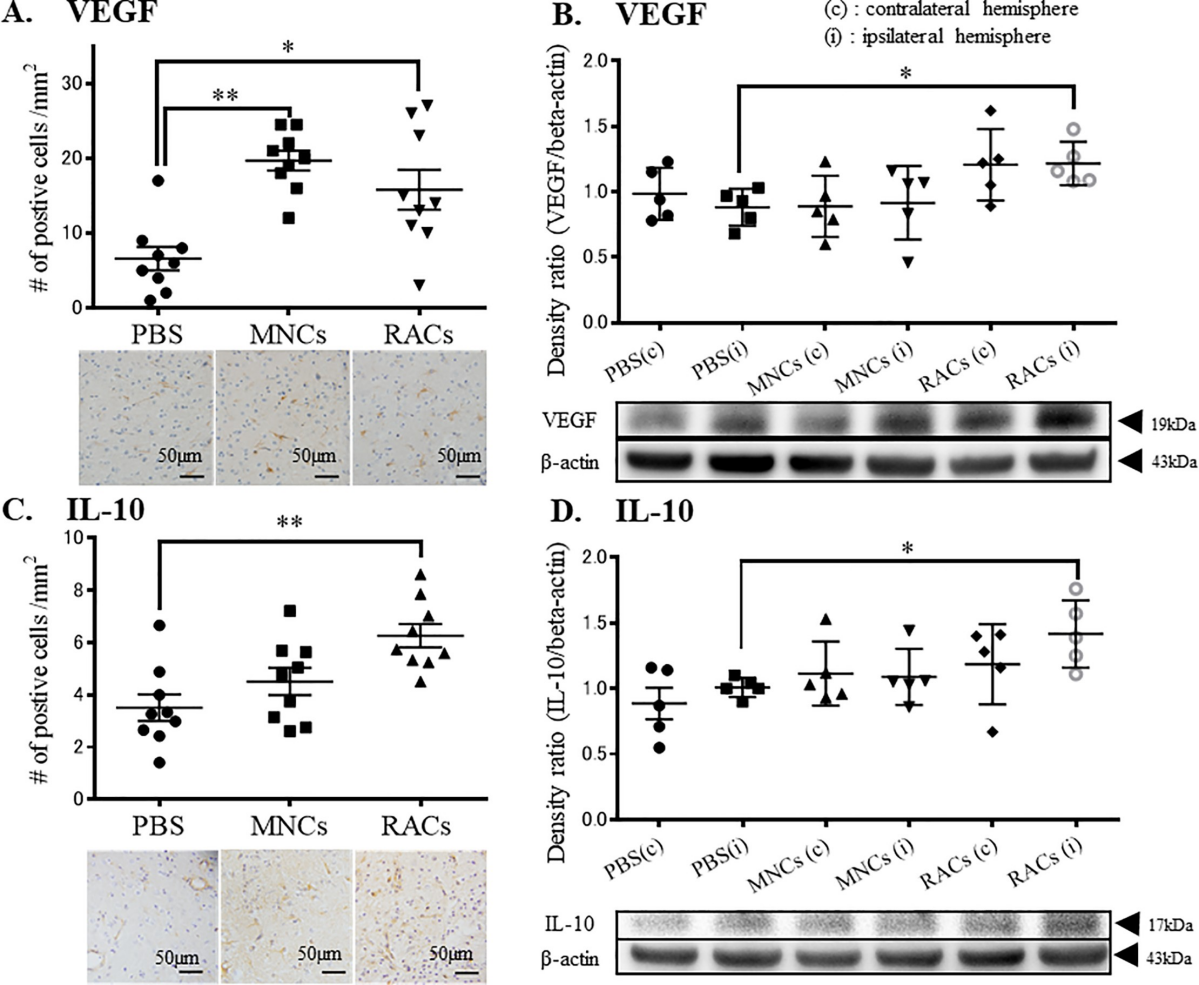
Immunohistochemistry and western blotting of ipsilateral hemisphere tissue at day 7. (A) Injection of either MNCs or RACs significantly increased VEGF-positive cells in the peri-infarct area at day 7 (each group: n = 9) (**P<0.01 and *P<0.05, respectively, Kruskal-Wallis test). Scale bar: 50 μm. (B) Injection of RACs (1 x 10^4^/50 μL) significantly increased VEGF in the peri-infarct area, as determined by western blotting, at day 7 (*P<0.05, Kruskal-Wallis test). (PBS: n = 6, MNCs: n = 6, RACs: n = 7) (C) Injection of RACs (1 x 10^4^ /50 μL) significantly increased IL-10-positive cells in the peri-infarct area at day 7 (*P<0.05, Kruskal-Wallis test) (each group: n = 9). Scale bar: 50 μm. (D) Injection of RACs (1 x 10^4^ /50 μL) significantly increased IL-10 in the peri-infarct area, as determined by western blotting, at day 7 (*P<0.05, Kruskal-Wallis test) (PBS: n = 6, MNCs: n = 6, RACs: n = 7).

**Fig 6:**
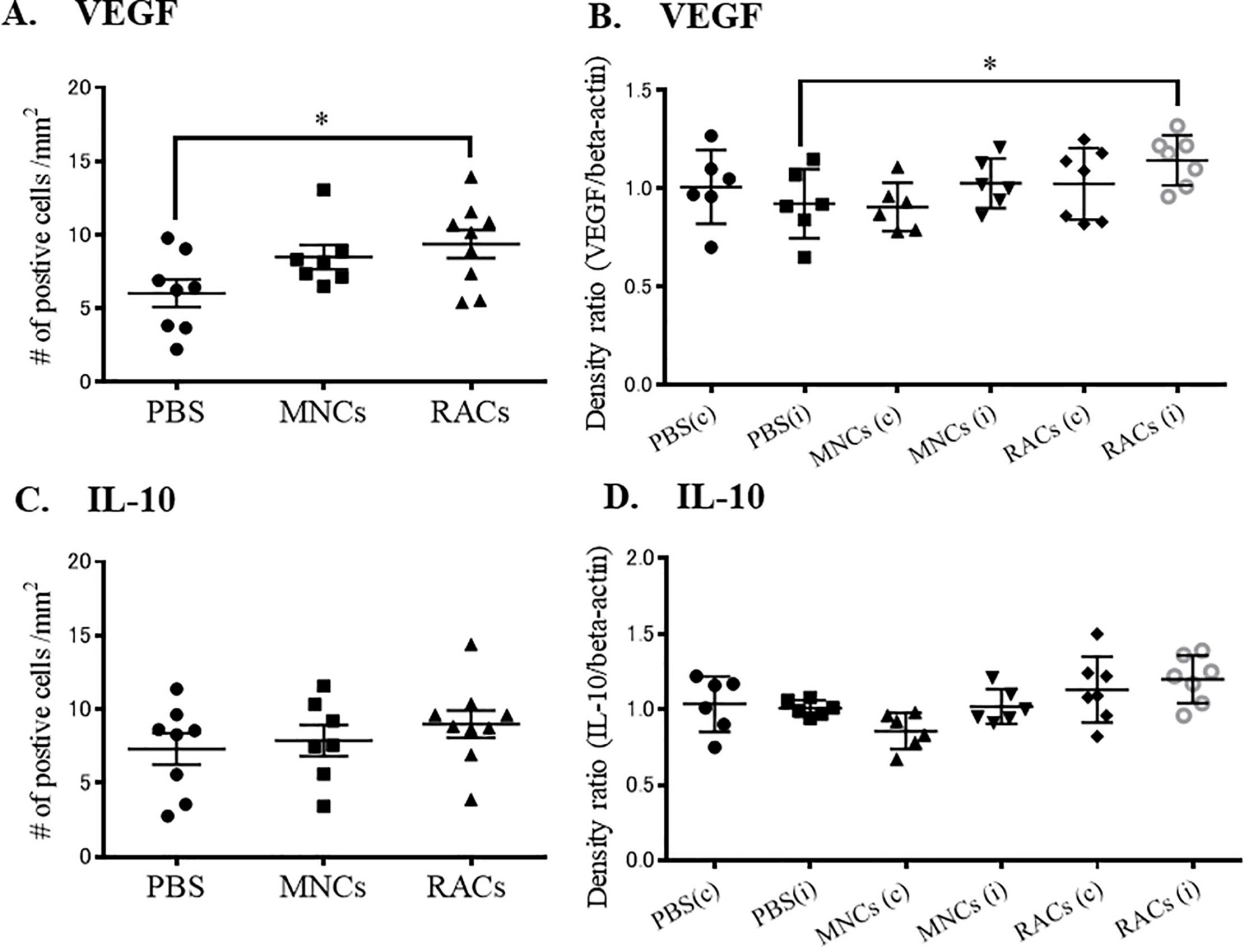
Immunohistochemistry and western blotting of ipsilateral hemisphere tissue at day 21. (A) Injection of either MNCs or RACs significantly increased VEGF-positive cells in the peri-infarct area at day 21 (PBS: n = 8, MNCs: n = 7, RACs: n = 9) (*P<0.05, respectively, Kruskal-Wallis test). (B) Injection of RACs (1 x 104/50 μL) significantly increased VEGF in the peri-infarct area, as determined by western blotting, at day 21 (*P<0.05, Kruskal-Wallis test) (PBS: n = 6, MNCs: n = 6, RACs: n = 7). (C) Injection of RACs (1 x 104 /50 μL) did not increase IL-10-positive cells in the peri-infarct area at day 21 (PBS: n = 8, MNCs: n = 7, RACs: n = 9). (D) Injection of RACs (1 x 10^4^ /50 μL) did not increase IL-10 in the peri-infarct area, as determined by western blotting, at day 21 (PBS: n = 6, MNCs: n = 6, RACs: n = 7).

To evaluate the effect of RAC transplantation on severe ischemia tissues in MCAO, we examined the expression profiles of anti-inflammatory cytokine, IL-10, and angiogenesis factor, VEGF, at the early phase of recovery (day 7) in the peri-infarct area. RAC-transplanted animals at day 7 showed increased cell numbers of both VEGF and IL-10 expressing cells, determined by immunohistochemistry (Fig 5A and 5C). These findings were supported by the results of western blotting, which showed increased expression of IL-10 and VEGF protein in the ipsilateral hemisphere at day 7 (Fig 5B and 5D). Late phase recovery tissue at day 21 was also investigated, and showed an increase of VEGF, but not IL-10 (Fig 6A, 6B, 6C and 6D).

### Neovascularization of leptomeningeal collateral arteries with RACs

**Fig 7:**
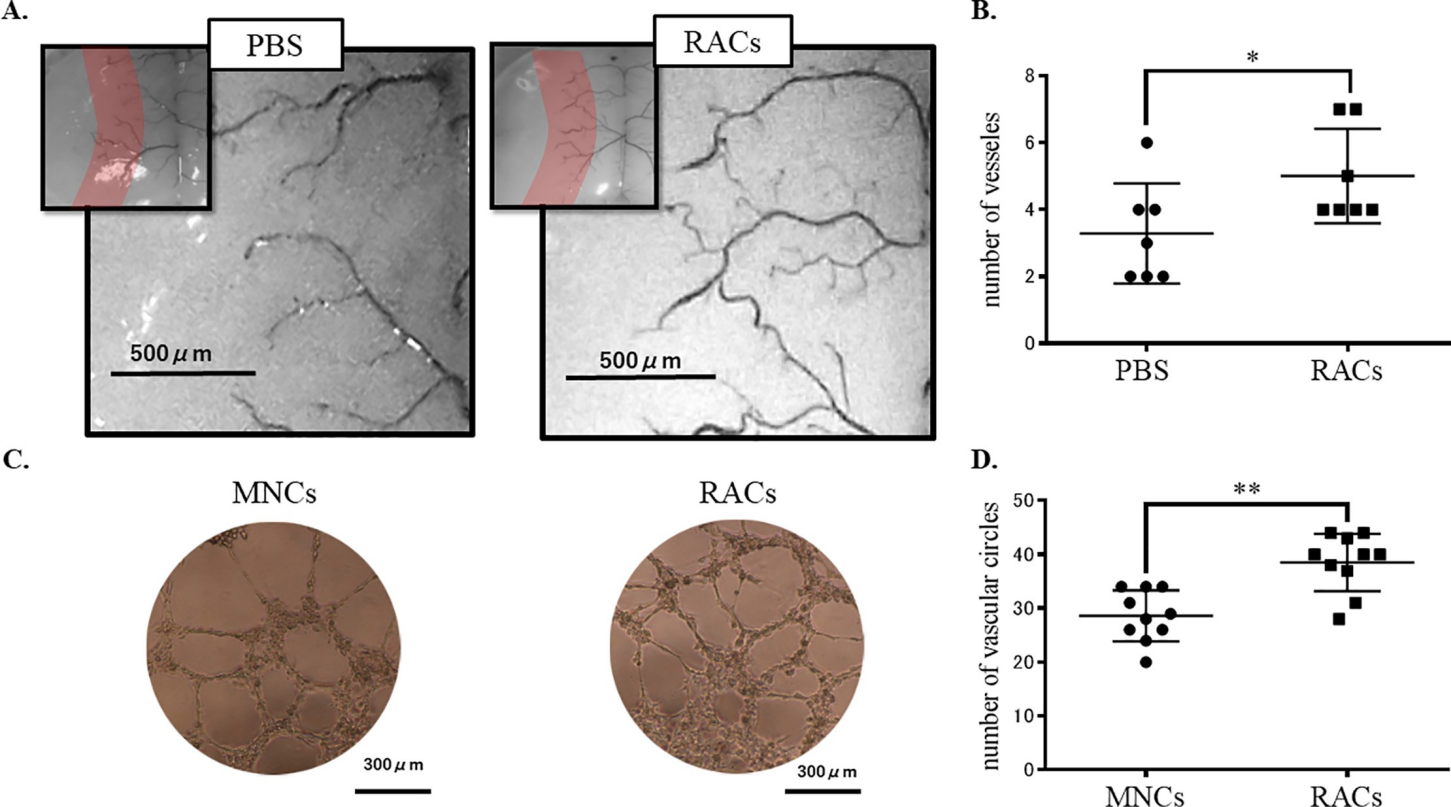
Perfusion of ink mixed with urethane resin and Matrigel tube formation assay. Capillary-like tubes were observed microscopically in tube formation assay. The number of vascular circles in the RACs group was significantly higher than that in the MNCs group (n = 7 each group) (*P<0.05, Mann Whitney test). The number of blood vessels in a pre-determined area extending 0.6 mm from the edge of MCA territory was examined. The number of arteries at the surface of the brain in the boundary zone was significantly increased in RACs (1 x 10^4^ /50 μL)-treated mice at day 7 (n = 10 for each group) (**P<0.01, Mann-Whitney).

To evaluate the effect of RAC transplantation on perfusion recovery, ink perfusion assay was applied. The number of detected arteries at the surface of the brain in the boundary zone between MCA territory and the anterior/ posterior cerebral artery (Fig 7A) was significantly increased (P<0.05) in mice treated with RACs at day 1 and sacrificed at day 7, compared with the PBS-injected group (Fig 7B).

**Fig 8:**
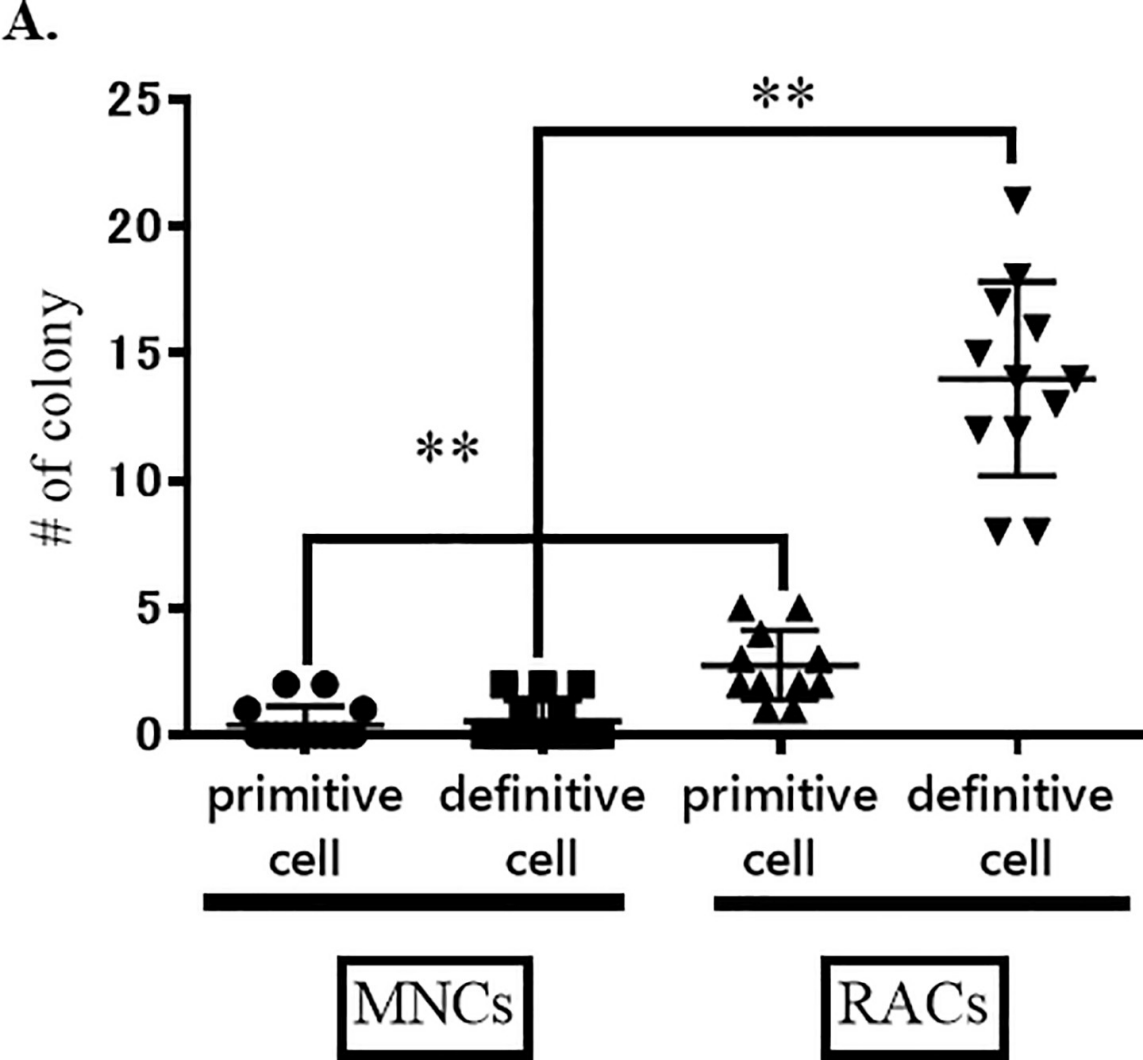
MNCs and RACs colony assay. The numbers of primitive and definitive cell colonies after injection of RACs (n = 15) were significantly increased (**P<0.01) at day 7, as compared with those after injection of MNCs (n = 12).

In vitro Matrigel tube formation assay supported the in vivo blood vessel formation results, showing a significantly increased number of vascular circles in the RAC treated group compared to the MNC treated group (P<0.01, Mann Whitney test, Fig 7C and 7D). EPC Colony-forming assay results also confirmed enhanced vasculogenic activity after RAC administration; there was a significant increase of definitive EPC colonies in RACs, compared with MNCs (Fig 8). These results indicate that RACs strongly promote neovascularization, compared to MNCs.

## Discussion

Our results indicate that RACs, produced by simple vasculogenic conditioning of MNCs could be a potent, safe and convenient cell population for promoting recovery after severe cerebral ischemia, based on their anti-inflammatory and angiogenic effects.

There is a great deal of evidence showing that EPCs promote recovery after ischemic cerebral injury [4–10, 23, 24], but EPCs are difficult to prepare in quantity and are expensive. EPCs were first isolated from peripheral blood (PB) of adults in 1997 [11], and circulating EPCs derived from bone marrow were shown to contribute to postnatal physiological and pathological neovascularization.[12, 13] With the aim of obtaining EPC-enriched cell populations for clinical application with a simple and effective culture system, we have recently developed a novel cell population, named regeneration-associated cells (RACs), by means of quality- and quantity-controlled culture of unfractionated mononuclear cells (MNCs) [14, 15] in the presence of human recombinant stem cell factors. The methodology is simple and safe, and can be used not only to enhance EPC expansion, but also to activate anti-inflammatory and angiogenic monocytes/macrophages and helper T lymphocytes, enabling the delivery of various protective and proangiogenic cytokines and growth factors. [14, 15] RACs contain expanded populations of progenitor cells (CD34^+^ or CD133^+^ cells) and anti-inflammatory monocytes and macrophages (CD206^+^ cells). [15] The increase of CD34^+^ or CD133^+^ cells indicates an expanded population of primitive EPCs, while the increase in CD105^+^ or CD146^+^ cells suggests EPC expansion and differentiation. [15] Further, the increase of CD206^+^ cells and decrease of CCR2^+^ cells indicate conversion of the monocyte/macrophage phenotype from M1 to M2. Monocytes/macrophages differentiate toward a pro-inflammatory, classically activated M1 state or toward an anti-inflammatory, alternatively activated M2 state in response to different environments and stimuli. M2 macrophages are induced by anti-inflammatory cytokines, such as IL-4, IL-10, and IL-13, and they ameliorate type 1 inflammatory responses and control adaptive immunity. [14, 15] Furthermore, their secreted anti-inflammatory cytokines promote and regulate type 2 immune responses, angiogenesis, and tissue repair. Thus, monocyte/macrophages in RACs mainly exhibit angiogenic and anti-inflammatory phenotypes, and are expected to contribute to the regenerative process in ischemic stroke.

The ischemic tissues in peri-infarcted area in acute phase of cerebral infarction are dominated by inflammation and early angiogenesis to trigger tissue regeneration. However, excessive inflammation in severe ischemia causes undesired destruction of tissues and prevents angiogenesis and tissue recovery. Post-ischemic inflammation can appear from hours to days after cerebral ischemia in humans [25, 26]. Brain ischemia triggers the secretion of damage-associated molecules, such as the chromatin-associated protein termed high mobility group protein B1, heat-shock proteins, ATP, S100 proteins, heparan sulfate, DNA, and RNA [27]. These molecules trigger a sterile inflammatory response characterized by an orchestrated infiltration of the brain parenchyma by immune cells, according to a temporal pattern coordinated and upregulated by chemoattractants and adhesion molecules. [28, 29] Since RACs can suppress neuro-inflammation through upregulation of IL-10, it is plausible that RACs administration at 1 to 3 days after cerebral ischemia in mice would be associated with a favorable outcome. This conclusion is consistent with a previous study [30] suggesting that neural stem cells should be transplanted in the early post-stroke phase, before the inflammatory response is established.

Several possible mechanisms of neuroprotection and arteriogenesis by RACs can be considered. RACs increased IL-10- and VEGF-positive cells at day 7. VEGF is known to play a key role in initiating physiological and pathological angiogenesis through VEGF/VEGF receptors system and mediating the development and growth of collateral vessels after ischemic stroke.[31] Marti et al. [32] reported that VEGF was strongly up-regulated, accompanied with an increase in the number of newly formed vessels in the ischemic border, within 6 to 24 hours after MCAO, while VEGF receptors were up-regulated at 48 hours and later in the ischemic core. Another report suggested that administration of VEGF decreased infarct area, reduced cell apoptosis and stimulated angiogenesis in mice with ischemic stroke [33]. On the other hand, the multifunctional cytokine IL-10 has anti-inflammatory and immune-regulatory effects.[34] Several studies have shown that IL-10 has antiangiogenic properties, though the effect of IL-10 on angiogenesis is controversial [35–38]; however, it appears that IL-10 can have proangiogenic and anti-inflammatory properties at least under some conditions [39–43] Notably, focal and systemic administrations of IL-10 significantly decreased infarct size in permanent focal ischemia.[44]

These findings of IL-10 and VEGF involvement in tissue recovery are supported by various findings in the field of cardiovascular medicine. Kishore’s group established a cardioprotective role of IL-10 in mouse models of acute myocardial infarction and pressure-overloaded myocardium. [43, 45]　 IL-10 knockout (IL-10KO) mice have been used as a model of systemic inflammation, and it was found that EPCs from IL-10 KO mice have diminished ability to facilitate angiogenesis and post-myocardial infarction recovery. [43, 45] These findings support the idea that IL-10 plays a significant role in EPC-related angiogenesis. It is possible that transplantation of RACs including EPCs and M2 macrophages contributes to microenvironmental regulation to induce IL-10-mediated antiinflammation and VEGF-mediated vascularization in brain ischemia, although further work will be needed to confirm this.

We observed an increase in the number of VEGF-positive cells at the boundary zone, but not at the core of infarction, at days 7 and 21. Western blotting also showed an increase in VEGF expression at day 7, and latex perfusion assay indicated that the number of arteries at the boundary zone was significantly increased at day 7. These results demonstrate that intra-arterial transplantation of RAC induces arteriogenesis through upregulation of VEGF at the boundary zone, but not at the core of infarct.

As regards the potential clinical application of RACs to brain ischemia patients, it should be noted that the infarct volume depends on neuronal death and edema, which are mainly associated with inflammation, especially during the acute phase. RACs produce anti-inflammatory responses through immune-mediators including IL-10 and VEGF, so RACs might reduce the infarct volume in post-ischemic stroke in humans, too. For clinical use, RACs could be produced within 5 days by culture, so RACs could be administered in the acute or subacute phase in clinical practice. In the chronic phase, the infarct volume depends on tissue clearance and neurogenesis, which is correlated with angiogenesis. RACs could also enhance angiogenesis in the acute or subacute phase, because the ink perfusion study and Matrigel tube formation assay showed increased numbers of vessels in the peri-infarct region and enhanced vasculogenic activity. Thus, in the chronic phase, RACs could provide a vascular-rich environment, which would favor tissue clearance and neurogenesis. Although we observed a significant decrease in infarct volume in RACs-treated mice, we did not detect a difference in functional recovery between RAC-treated and control animals. The reason for this might be that the standard recovery from neurological deficit in this mouse model is too rapid. It is well known that the permanent MCAO model in mice causes morphological damage, but recovery of physical status generally occurs within a week. Further studies using a different stroke model or MCAO in a different strain, or large animals (primates) should be considered in order to see whether or not RACs speed up functional recovery from neurological deficits.

## Conclusion

Transplantation of RACs through the ipsilateral external carotid artery promoted recovery and regeneration of neurovascular units in mice with permanent occlusion of the MCA. Since RACs are enriched in cells with anti-inflammation and angiogenesis functionality through serum-free culture of cells obtained from a small sample of blood, RACs are expected to have potential for clinical treatment of cerebral ischemia by promoting angiogenesis and tissue regeneration.

## Supporting information

S1 Table NC3Rs ARRIVE guidelines checklist. (DOCX)

S1 File The values behind the means, standard deviations and standard errors for all data The means, SD and SE for Figs 2, 4, 5, 6, 7 and 8. (XLSX)
